# Supplementary material for: Impact of Post-Harvest Apple Scab on Peel Microbiota, Fermentation Dynamics, and the Volatile/Non-Volatile Composition of Cider
Source: Molecules. 2025 May 26;30(11):2322. doi: 10.3390/molecules30112322 (PMC12156036; doi:10.3390/molecules30112322)
Supplement: Supplementary file 1 [file molecules-30-02322-s001.zip › molecules-3589122-supplementary.pdf]

SUPPLEMENTARY MATERIAL

Table S1. The physicochemical parameters of apples measured after the cold storage with an automatic fruit-sorting analyser (Pimprenelle; Setop Giraud Technology, Cavaillon, France). Data are the average of the measure of 24 apples for each sample. Within each cultivar no significant differences were present between healthy and scab-affected (RT) fruits.

**Apple sample**	**Total polyphenols** *mg/kg*	**Weight** *g*	**Sugars** *°Brix*	**Hardness** *kg/cm²*	**Acidity** *g/L*	**Juiciness** %
Golden healthy	504	193	14.2	4.7	3.1	10.7
Golden scab-affected	508	176	14.3	4.4	2.5	10.2
Gala healthy	714	159	12.8	7.1	3.2	9.9
Gala scab-affected	745	160	13.1	6.9	2.9	9.7

Table S2A. Relative abundance of bacterial taxa identified by Greengenes.

**Phylum**	**Taxon**	**Rank**	**Relative amount of total (%)**
[Thermi]	*Deinococcus*	Genus	0.134
[Thermi]	*Deinococcus aquatilis*	Species	0.019
[Thermi]	*Truepera*	Genus	0.002
Acidobacteria	*Ellin6075*	Family	0.049
Actinobacteria	*Acidimicrobiales*	Order	0.007
Actinobacteria	*Aeromicrobium*	Genus	0.229
Actinobacteria	*Arthrobacter*	Genus	0.047
Actinobacteria	*Brevibacterium aureum*	Species	0.075
Actinobacteria	*Curtobacterium*	Genus	1.085
Actinobacteria	*Frigobacterium*	Genus	1.982
Actinobacteria	*Geodermatophilaceae*	Family	1.056
Actinobacteria	*Kineococcus*	Genus	0.779
Actinobacteria	*Kineosporiaceae*	Family	0.012
Actinobacteria	*Microbacteriaceae*	Family	2.276
Actinobacteria	*Micrococcaceae*	Family	0.008
Actinobacteria	*Nocardioidaceae*	Family	0.685
Actinobacteria	*Patulibacter*	Genus	0.031
Actinobacteria	*Rhodococcus*	Genus	0.030
Actinobacteria	*Rhodococcus fascians*	Species	1.881
Actinobacteria	*Solirubrobacterales*	Family	0.028
Actinobacteria	*Willamsia*	Genus	0.344
Armatimonadates	*Fimbriimonas*	Genus	0.025
Bacteroidetes	*Adhaeribacter*	Genus	0.039
Bacteroidetes	*Candidatus Cardinium*	Species	0.014
Bacteroidetes	*Chitinophaga*	Genus	0.010

Bacteroidetes	*Chitinophagaceae*	Family	0.132
Bacteroidetes	*Chryseobacterium*	Genus	0.708
Bacteroidetes	*Cytophagaceae*	Family	0.073
Bacteroidetes	*Dyadobacter*	Genus	0.283
Bacteroidetes	*Dysgonomonas*	Genus	0.003
Bacteroidetes	*Flavobacterium*	Genus	1.335
Bacteroidetes	*Flavobacterium succinicans*	Species	0.008
Bacteroidetes	*Flectobacillus*	Genus	0.079
Bacteroidetes	*Hymenobacter*	Genus	5.987
Bacteroidetes	*Pedobacter*	Genus	0.453
Bacteroidetes	*Pedobacter cryoconitis*	Species	0.679
Bacteroidetes	*Prevotella*	Genus	0.005
Bacteroidetes	*Runella*	Genus	0.003
Bacteroidetes	*Saprospiraceae*	Family	0.001
Bacteroidetes	*Sediminibacterium*	Genus	0.009
Bacteroidetes	*Sphingobacteriaceae*	Family	0.441
Bacteroidetes	*Sphingobacteriales*	Order	0.013
Bacteroidetes	*Spirosoma*	Genus	0.842
Bacteroidetes	*Sporocytophaga*	Genus	0.003
Chlamidiae	*Candidatus Protochlamydia*	Species	0.002
Chlamidiae	*Parachlamydiaceae*	Family	0.002
Chloroflexi	*A4b*	Family	0.007
Cyanobacteria	*Chlorophyta*	Order	0.087
Cyanobacteria	*Streptophyta*	Order	1.985
Cyanobacteria	*Xenococcaceae*	Family	0.015
FBP	*FBP*	Phylum	0.013
Firmicutes	*Alloiococcus*	Genus	0.010
Firmicutes	*Bacillus*	Genus	0.010
Firmicutes	*Bacillus flexus*	Species	0.019
Firmicutes	*Clostridiales*	Order	0.001
Firmicutes	*Clostridium*	Genus	0.012
Firmicutes	*Desemzia*	Genus	0.021
Firmicutes	*Enterococcus*	Genus	0.043
Firmicutes	*Exiguobacterium*	Genus	0.006
Firmicutes	*Lactobacillus*	Genus	0.029
Firmicutes	*Lactococcus*	Genus	0.047
Firmicutes	*Leuconostoc*	Genus	0.003
Firmicutes	*Leuconostoc mesenteroides*	Species	0.014
Firmicutes	*Staphilococcus epidermidis*	Species	0.063
Firmicutes	*Staphylococcus*	Genus	0.018
Firmicutes	*Trichococcus*	Genus	0.061
Firmicutes	*Weissella*	Genus	0.030
Planctomycetes	*Gemmata*	Genus	0.011
Planctomycetes	*Pirellulaceae*	Family	0.012
Proteobacteria	*Acetobacteraceae*	Family	1.457
Proteobacteria	*Acetobacteraceae*	Family	0.236
Proteobacteria	*Acinetobacter*	Genus	0.023
Proteobacteria	*Agrobacterium*	Genus	2.420

Proteobacteria	*Aurantimonadaceae*	Family	0.065
Proteobacteria	*Bdellovibrio*	Genus	0.003
Proteobacteria	*Bradyrhizobium*	Genus	0.012
Proteobacteria	*Buchnera*	Genus	0.012
Proteobacteria	*Caulobacteraceae*	Family	0.216
Proteobacteria	*Comamonadaceae*	Family	2.000
Proteobacteria	*Enterobacteriaceae*	Family	0.362
Proteobacteria	*Erwinia*	Genus	3.429
Proteobacteria	*Erythrobacteraceae*	Family	0.038
Proteobacteria	*Gluconobacter*	Genus	4.282
Proteobacteria	*Gluconobacter*	Genus	0.100
Proteobacteria	*Gluconobacter*	Genus	0.005
Proteobacteria	*Janthinobacterium*	Genus	2.184
Proteobacteria	*Janthinobacterium lividum*	Species	0.101
Proteobacteria	*Methylocystaceae*	Family	0.506
Proteobacteria	*Metylobacterium*	Genus	4.489
Proteobacteria	*Metylobacterium adhaesivum*	Species	4.317
Proteobacteria	*mithocondria*	Family	0.011
Proteobacteria	*Mixococcales*	Order	0.010
Proteobacteria	*Mycoplana*	Genus	0.025
Proteobacteria	*Novosphingobium*	Genus	0.052
Proteobacteria	*Oxalobacteraceae*	Family	6.413
Proteobacteria	*Oxalobacteraceae*	Family	0.003
Proteobacteria	*Paracoccus marcusii*	Species	0.163
Proteobacteria	*Pasteurellales*	Family	0.096
Proteobacteria	*Pseudomonas*	Genus	3.072
Proteobacteria	*Pseudomonas*	Genus	0.006
Proteobacteria	*Pseudomonas veronii*	Species	0.036
Proteobacteria	*Pseudomonas viridiflava*	*Species*	*10.287*
Proteobacteria	*Psychobacter pulmonis*	Species	0.005
Proteobacteria	*Psychrobacter*	Genus	0.030
Proteobacteria	*Psychrobacter celer*	Genus	0.012
Proteobacteria	*Rhodoplanes elegans*	Species	0.002
Proteobacteria	*Rickettsiales*	Family	0.004
Proteobacteria	*Sinobacteraceae*	Family	0.121
Proteobacteria	*Skermanella*	Genus	0.113
Proteobacteria	*Sphingobium*	Genus	0.675
Proteobacteria	*Sphingomonadaceae*	Family	1.982
Proteobacteria	*Sphingomonas*	Genus	21.136
Proteobacteria	*Sphingomonas echinoides*	Species	0.065
Proteobacteria	*Sphingomonas wittichii*	Species	0.868
Proteobacteria	*Spirobacillales*	Order	0.004
Proteobacteria	*Thermomonas*	Genus	0.010
Proteobacteria	*Variovorax*	Genus	3.078
Proteobacteria	*Xanthomonadaceae*	Family	0.867
Proteobacteria	*Zoogloea*	Genus	0.005
TM7	*EW055*	Order	0.122

Table S2B. Relative abundance of fugal taxa identified by UNITE

Phylum	Taxon	Rank	Relative amount of total (%)
Ascomycota	*Acremonium*	Genus	0.002
Ascomycota	*Acremonium alternatum*	Species	2.196
Ascomycota	*Acremonium fusidioides*	Species	0.111
Ascomycota	*Acremonium pilosum*	Species	0.074
Ascomycota	*Alternaria*	Genus	5.103
Ascomycota	*Amphisphaeriaceae*	Family	0.007
Ascomycota	*Angustimassarina acerina*	Species	0.007
Ascomycota	*Articulospora*	Genus	0.096
Ascomycota	*Ascomycota*	Phylum	2.178
Ascomycota	*Aspergillus*	Genus	0.001
Ascomycota	*Aspergillus conicus*	Species	0.004
Ascomycota	*Aureobasidium pullulans*	Species	3.509
Ascomycota	*Bipolaris*	Genus	0.001
Ascomycota	*Buellia frigida*	Species	0.002
Ascomycota	*Cadophora*	Genus	0.020
Ascomycota	*Candida*	Genus	0.065
Ascomycota	*Candida norvegica*	Species	0.018
Ascomycota	*Candida sake*	Species	0.052
Ascomycota	*Capnodiales*	Order	0.095
Ascomycota	*Ceratocystidaceae*	Family	0.021
Ascomycota	*Chaetosphaeronema*	Genus	0.002
Ascomycota	*Chitinophaga*	Genus	0.006
Ascomycota	*Cladosporium*	Genus	12.119
Ascomycota	*Cladosporium sphaerospermum*	Species	0.219
Ascomycota	*Cyberlindnera misumaiensis*	Species	0.001
Ascomycota	*Cyphellophora reptans*	Species	0.003
Ascomycota	*Devriesia pseudoamericana*	Species	0.045
Ascomycota	*Didymella fabae*	Species	0.068
Ascomycota	*Didymella urticicola*	Species	0.017
Ascomycota	*Didymellaceae*	Family	15.791
Ascomycota	*Didymosphaeriaceae*	Family	0.003
Ascomycota	*Diplodia allocellula*	Species	0.018
Ascomycota	*Dothideomycetes*	Class	0.014
Ascomycota	*Eremascus fertilis*	Species	0.001
Ascomycota	*Eucasphaeria*	Genus	0.008
Ascomycota	*Fusarium*	Genus	0.029
Ascomycota	*Fusarium solani*	Species	0.003
Ascomycota	*Genolevuria*	Genus	0.015
Ascomycota	*Graphium*	Genus	0.009
Ascomycota	*Hansfordia pulvinata*	Species	0.003
Ascomycota	*Helotiales_fam_Incertae_sedis*	Family	0.008
Ascomycota	*Hypocreales*	Order	0.240
Ascomycota	*Knufia*	Genus	0.001

Ascomycota	*Kregervanrija fluxuum*	Species	0.002
Ascomycota	*Lachneulla*	Genus	0.001
Ascomycota	*Leptosphaeria*	Genus	0.022
Ascomycota	*Leptosphaeria rubefaciens*	Species	0.527
Ascomycota	*Melanommataceae*	Family	0.144
Ascomycota	*Microcyclospora tardicrescens*	Species	0.004
Ascomycota	*Mycosphaerella tassiana*	Species	6.020
Ascomycota	*Mycospherellaceae*	Family	0.091
Ascomycota	*Nectriaceae*	Family	4.960
Ascomycota	*Nigrospora oryzae*	Species	0.002
Ascomycota	*Paraconiothyrium africanum*	Species	0.001
Ascomycota	*Paraphoma*	Genus	0.009
Ascomycota	*Penicilium*	Genus	0.422
Ascomycota	*Penicillium bialowiezense*	Species	0.075
Ascomycota	*Penicillium camemberti*	Species	0.001
Ascomycota	*Periconia*	Genus	0.006
Ascomycota	*Phaeosphaeria*	Genus	0.001
Ascomycota	*Phaeosphaeriaceae*	Family	0.176
Ascomycota	*Plectosphaerella cucumerina*	Species	0.032
Ascomycota	*Pleosporaceae*	Family	0.010
Ascomycota	*Pleosporales*	Order	0.007
Ascomycota	*Pleurophoma ossicola*	Species	0.022
Ascomycota	*Radulidium subulatum*	Species	0.017
Ascomycota	*Ramularia*	Genus	12.240
Ascomycota	*Sarocladium strictum*	Species	0.034
Ascomycota	*Sclerostagonospora cycadis*	Species	0.003
Ascomycota	*Sclerotiniaceae*	Family	0.239
Ascomycota	*Stachybotryaceae*	Family	0.005
Ascomycota	*Stemphylium*	Genus	0.003
Ascomycota	*Stibella*	Genus	0.531
Ascomycota	*Taphrina*	Genus	0.034
Ascomycota	*Taphrina tormentillae*	Species	0.072
Ascomycota	*Tetracladium*	Genus	0.002
Ascomycota	*Trichoderma*	Genus	0.002
Ascomycota	*Venturia asperata*	Species	0.001
Ascomycota	*Venturiales*	Order	0.001
Ascomycota	*Wickerhamomyces anomalus*	Species	0.002
Ascomycota	*Xylariales*	Order	0.002
Ascomycota	*Zymoseptoria*	Genus	1.577
Basidiomycota	*Acaromyces ingoldii*	Species	0.007
Basidiomycota	*Agaricomycetes*	Class	0.029
Basidiomycota	*Anthracocystis*	Genus	0.001
Basidiomycota	*Basidiomycota*	Phylum	2.067
Basidiomycota	*Bensingtonia*	Genus	0.005
Basidiomycota	*Buckleyzyma aurantiaca*	Species	0.323
Basidiomycota	*Bulleromyces*	Genus	0.026
Basidiomycota	*Chionosphaeraceae*	Family	0.002
Basidiomycota	*Cryptococcus uniguttulatus*	Species	0.036

Basidiomycota	*Cystobasidiomycetes*	Class	0.792
Basidiomycota	*Cystobasidium*	Genus	0.005
Basidiomycota	*Cystobasidium pinicola*	Species	1.042
Basidiomycota	*Cystofilobasidiales*	Order	0.791
Basidiomycota	*Cystofilobasidium capitatum*	Species	0.265
Basidiomycota	*Cystofilobasidium infirmominiatum*	Species	0.006
Basidiomycota	*Dioszegia*	Genus	0.042
Basidiomycota	*Entyloma*	Genus	0.084
Basidiomycota	*Entylomatales*	Order	0.673
Basidiomycota	*Erythrobasidium hasegawianum*	Species	0.046
Basidiomycota	*Exobasidium*	Genus	0.060
Basidiomycota	*Exobasidium maculosum*	Species	0.007
Basidiomycota	*Filobasidium*	Genus	0.058
Basidiomycota	*Filobasidium globisporum*	Species	1.350
Basidiomycota	*Filobasidium magnum*	Species	1.753
Basidiomycota	*Filobasidium wieringae*	Species	0.002
Basidiomycota	*Golubevia pallescens*	Species	0.002
Basidiomycota	*Guehomyces pullulans*	Species	0.081
Basidiomycota	*Hannaella*	Genus	0.001
Basidiomycota	*Holtermanniella takashimae*	Species	0.125
Basidiomycota	*Leucospiridium*	Genus	0.023
Basidiomycota	*Leucosporidium fellii*	Species	0.256
Basidiomycota	*Leucosporoidiales*	Order	0.020
Basidiomycota	*Naganishia albida*	Species	0.470
Basidiomycota	*Rhodotorula graminis*	Species	0.053
Basidiomycota	*Sporidiobolus*	Genus	0.011
Basidiomycota	*Sporobolomyces*	Genus	0.003
Basidiomycota	*Sporobolomyces patagonicus*	Species	0.011
Basidiomycota	*Symmetrospora coprosmae*	Species	0.974
Basidiomycota	*Tilletiopsis washingtonensis*	Species	1.451
Basidiomycota	*Tremellomycetes*	Class	0.019
Basidiomycota	*Trichosporonaceae*	Family	0.029
Basidiomycota	*Uncobasidium*	Genus	0.002
Basidiomycota	*Vanrija fragicola*	Species	0.002
Basidiomycota	*Vishniacozyma*	Genus	1.501
Basidiomycota	*Vishniacozyma carnescens*	Species	5.608
Basidiomycota	*Vishniacozyma dimennae*	Species	0.133
Basidiomycota	*Vishniacozyma victoriae*	Species	8.294
Basidiomycota	*Wallemia muriae*	Species	0.002
Blastocladiomycota	*Blastocladiales*	Order	0.003

Figure S1. Chromatograms of standards and a cider sample: (a) HPLC-FLD for aminoacid quantification; (b) HPLC-RI for lactic acid and glicerin; (c) IC-PAD for sugars; (d) IC-COND for organic acid analysis.

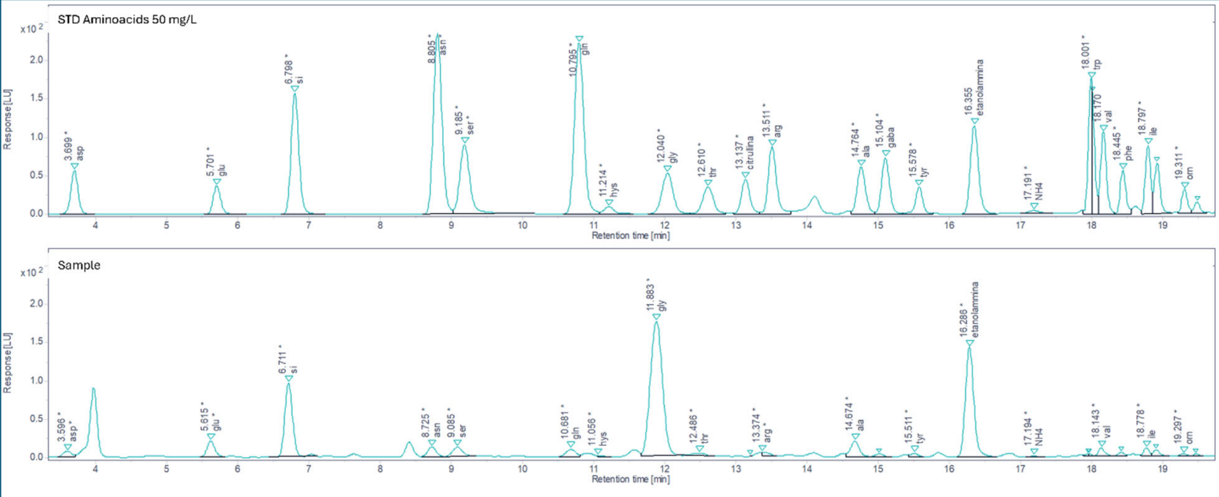

(a)

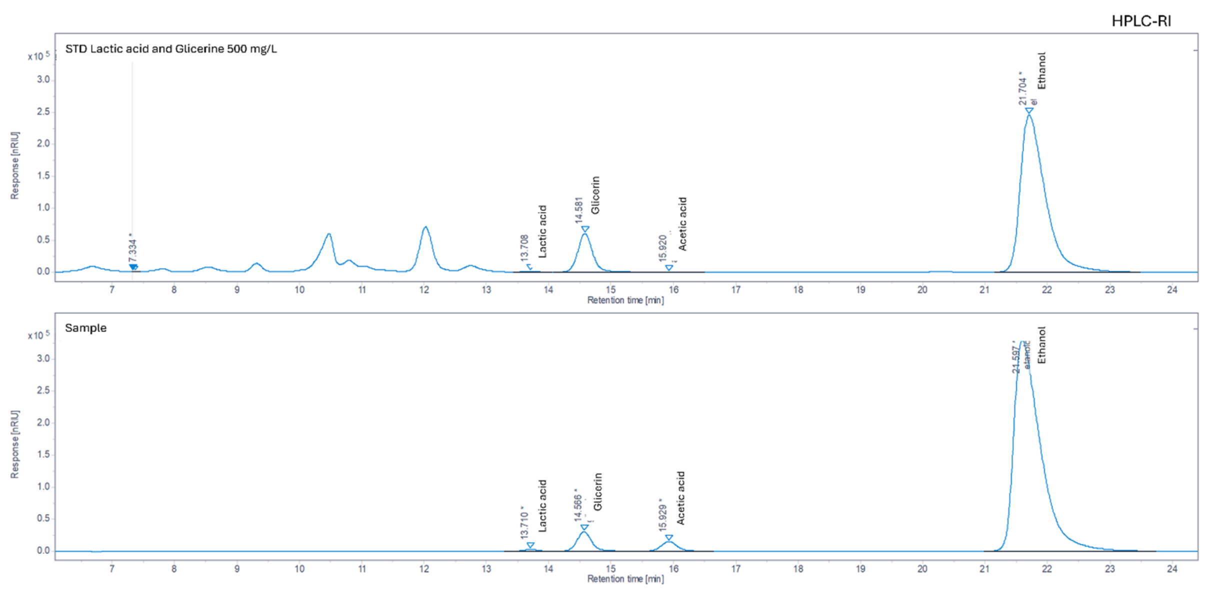

(b)

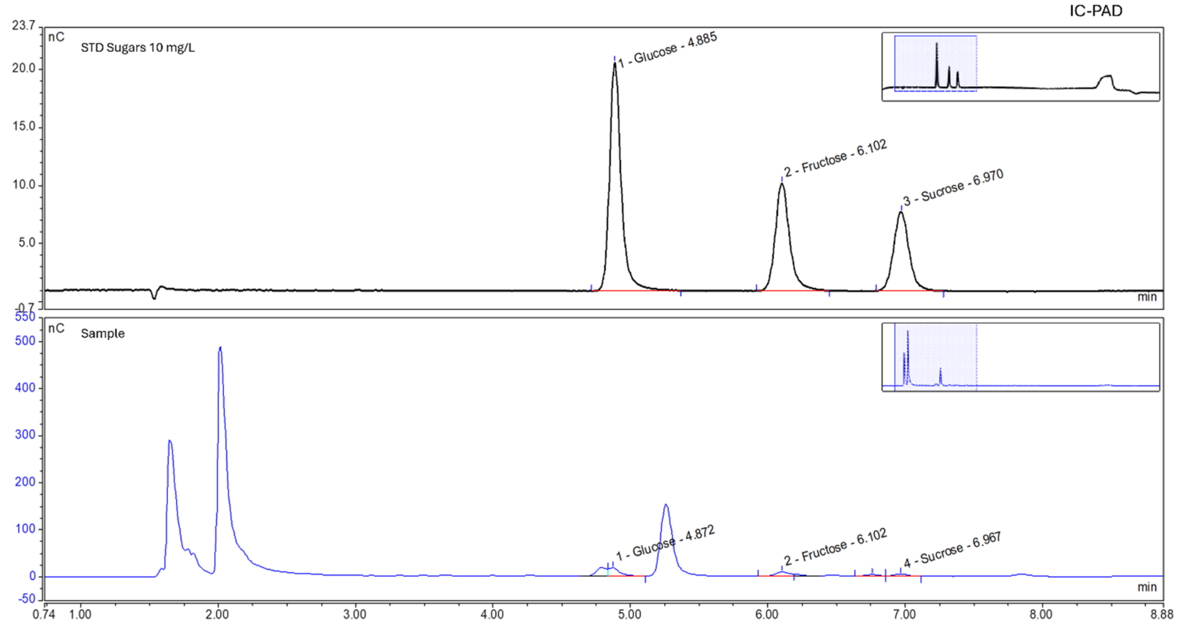
IC-PAD
nC
STD Sugars 10 mg/L
1 - Glucose - 4.885
2 - Fructose - 6.102
3 - Sucrose - 6.970
Sample
1 - Glucose - 4.872
2 - Fructose - 6.102
4 - Sucrose - 6.967
min

(c)

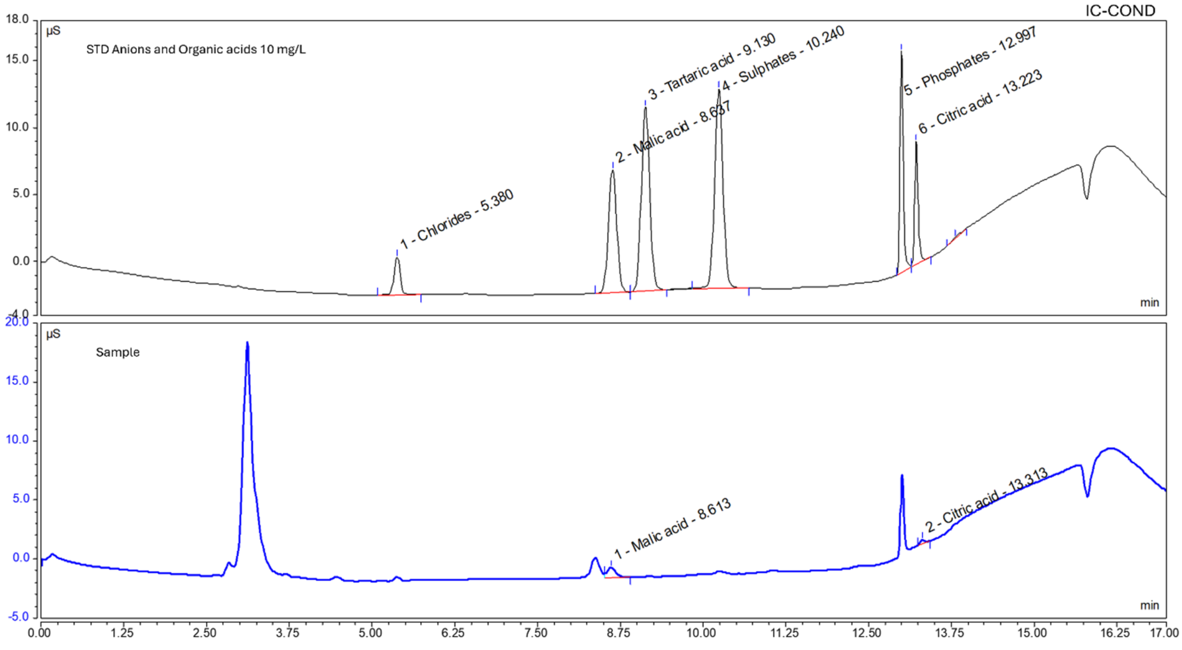
IC-COND
µS
STD Anions and Organic acids 10 mg/L
1 - Chlorides - 5.380
2 - Malic acid - 8.637
3 - Tartaric acid - 9.130
4 - Sulphates - 10.240
5 - Phosphates - 12.997
6 - Citric acid - 13.223
Sample
1 - Malic acid - 8.613
2 - Citric acid - 13.313
min

(d)
